# Behaviors of Organic Ligands and Phosphate during Biochar-Driven Nitrate Adsorption in the Presence of Low-Molecular-Weight Organic Acids

**DOI:** 10.3390/molecules27185811

**Published:** 2022-09-08

**Authors:** Wenming Xiong, Yongjun Li, Jidong Ying, Chuxia Lin, Junhao Qin

**Affiliations:** 1 Department, Guangdong Jiangmen Chinese Medicine College, Jiangmen 529000, China; 2 Key Laboratory of Agro-Environment in the Tropics, Ministry of Agriculture of China, College of Natural Resources and Environment, South China Agricultural University, Guangzhou 510642, China; 3 Centre for Regional and Rural Futures, Faculty of Science, Engineering and Built Environment, Deakin University, Burwood, VIC 3125, Australia

**Keywords:** biochar, low-molecular-weight organic acids, phosphate, nitrate, adsorption

## Abstract

A batch experiment was conducted to examine the behavior of nitrate, organic ligands, and phosphate in the co-presence of biochar and three common low-molecular-weight organic acids (LMWOAs). The results show that citrate, oxalate, and malate ions competed with nitrate ion for the available adsorption sites on the biochar surfaces. The removal rate of LMWOA ligands by the biochar via adsorption grew with increasing solution pH. The adsorbed divalent organic ligands created negatively charged sites to allow binding of cationic metal nitrate complexes. A higher degree of biochar surface protonation does not necessarily enhance nitrate adsorption. More acidic conditions formed under a higher dose of LMWOAs tended to make organic ligands predominantly in monovalent forms and failed to create negatively charged sites to bind cationic metal nitrate complexes. This could adversely affect nitrate removal efficiency in the investigated systems. LMWOAs caused significant release of phosphate from the biochar. The phosphate in the malic acid treatment tended to decrease over time, while the opposite was observed in the citric- and oxalic-acid treatments. This was caused by re-immobilization of phosphate in the former due to the marked increase in solution pH over time.

## 1. Introduction

Immobilization of nitrate in wastewater and fertilized soils is important for minimizing nitrogen pollution that causes eutrophication in open-water environments [1,2]. The use of sorbents to bind nitrate is one of the methods to achieve this goal [3]. Biochar produced from biomass pyrolysis is a cheap sorbent that has been proposed for uses in wastewater treatment and soil amendment/remediation [4,5]. However, most biochar materials are negatively charged due to their alkaline nature [6,7], which does not favor the adsorption of anionic nitrate. Yang et al. [8] found that only a very small amount of nitrate was adsorbed to the biochar surface though nitrate fixation tended to increase with increasing pyrolysis temperature of the biochar materials. Qin et al. [9] observed enhanced immobilization of nitrate by biochar in the presence of soluble calcium, which acted as a bridge to allow binding of nitrate by negatively charged biochar in the form of CaNO_3_^+^. Without the bridging effect from a divalent cation, protonation of biochar surfaces is required to allow biochar to retain anionic nitrate. Sanford et al. [10] demonstrated adsorption of nitrate by HCl-treated biochar. Heaney et al. [11] showed that nitrate could be removed by the biochar from the solutions in the presence of low-molecular-weight organic acids (LMWOAs), such as citric, oxalic, and malic acids, at a lower millimolar level. This suggests that LMWOAs are effective media for nitrate adsorption in aqueous solutions. More importantly, LMWOAs are major constituents in plant root exudates [12]. Therefore, this finding has implications for understanding nitrate mobility in rhizospheric soils.

Apart from being a cheap sorbent, biochar can also serve as a source of phosphorus [13,14,15]. Due to the alkaline nature of biochar, it is unlikely that biochar-borne phosphate is present in forms of aluminium or iron phosphate minerals [16,17,18]. Given the negatively charged status, pristine biochar is not capable of binding anionic phosphate ions via adsorption either. Therefore, phosphate contained in biochar can only be in either soluble forms or insoluble phosphates of divalent cations, such as calcium and magnesium [19,20]. Acidification of biochar upon reaction with LMWOAs could solubilize divalent metal phosphate, resulting in mobilization of biochar-borne phosphate minerals [21]. The liberation of phosphate from biochar into the treated wastewater is environmentally undesirable given that phosphate is also a eutrophication-causing nutrient [22,23]. In soils, the release of biochar-borne phosphate by LMWOAs in rhizosphere enables uptake of phosphorus by plant roots [24,25]. On the other hand, protonation of biochar surfaces that simultaneously takes place during LMWOA-driven acidification may favor adsorption of soluble phosphate ions, resulting in re-immobilization of the solution-borne phosphate. LMWOA-driven acidification could also solubilize biochar-borne Al and Fe hydroxides [26,27]. Additionally, it is therefore possible that the soluble phosphate can react with the dissolved Al and Fe to form insoluble aluminium and iron phosphates [28,29,30]. So far, the interactive effect of these possible processes on the behavior of phosphate in LMWOA–biochar systems has not been adequately understood. Furthermore, the dynamics of LMWOA-derived organic ligands during biochar-driven nitrate adsorption were not investigated in Heaney et al. [11]. Organic ligands could react with cations to form cation-organic ligand complexes or insoluble salts [31,32,33]. They can also compete with nitrate and phosphate ions for the available adsorption sites on the protonated biochar surfaces. Therefore, examining the behavior of the LMWOA-derived organic ligands is also important for understanding their roles in affecting the mobilization/immobilization of nitrate and phosphate in these reaction systems. The current work was intended to fill the above knowledge gaps.

## 2. Results

### 2.1. Variations in Eh, EC, and pH during the Period of Experiment

Eh ranged from 346 to 578 mV. After the 1 h reaction, Eh tended to be lower in the controls than in either the LMWOA or LMWOA-biochar treatments. Within the three controls, the no biochar control (C2) had lower Eh compared to the other two controls (C1 and C3) prior to the 72 h. However, the opposite pattern was observed at the 168 h. There was a trend showing that Eh decreased over time in the systems containing LMWOAs (Table 1).

The EC values in C1 (biochar only), C2 (nitrate only), and C3 (biochar and nitrate) were 0.33, 0.09, and 0.39 dS/m after the 1 h reaction, respectively. These levels were maintained with a slight fluctuation during the period of the experiment. The addition of LMWOAs increase the EC values compared to the controls with oxalic-acid treatment (TON) having a higher EC relative to the citric- and malic-acid treatments. The further addition of biochar (TOBN) resulted in a significant decrease in EC in the oxalic-acid treatment at any of the four measurement occasions during the experiment (Table 1).

The pH in C1 was 8.55 and increased over time to 9.82 at the 168 h. C2 had a circumneutral pH during the period of the experiment. The co-presence of nitrate and biochar (C2) resulted in a slight decrease in pH compared to C1, although it was not at a significant level. The addition of LMWOAs caused a decrease in pH with oxalic-acid treatment (TON) having a lower pH compared to the other organic-acid treatments. The further addition of biochar resulted in an increase in pH, and all three organic-acid treatments showed a trend that pH increased over time during the experiment. By comparison, pH was lower in the oxalic-acid treatment (TOBN) than in the other two organic-acid treatments at any of the four measurement occasions (Table 1).

### 2.2. Nitrite and Nitrate

No nitrite was detected in any controls and treatments. In C1 (biochar only), the nitrate concentration was 0.52 mg/L after the 1 h reaction. There was a trend of solution nitrate increasing over time. The concentration of nitrate in C2 (nitrate only) was more or less equivalent to the theoretical concentration of the added nitrate. In C3 (biochar and nitrate), the concentration of nitrate was higher after the 1 and 24 h and lower after the 72 and 168 h compared to C2. For the LMWOA treatments, the concentration of nitrate was also comparable to the theoretical concentration of the added nitrate except for the citric-acid treatment (TCN) at the 72 and 168 h. In the co-presence of LMWOA and biochar, the concentration of nitrate was reduced to below 30 mg/L from 62 mg/L (the added nitrate) after the 1 h reaction. No nitrate was detected on any of the later sampling occasions (Table 2).

### 2.3. Organic Ligands

**Figure 1:**
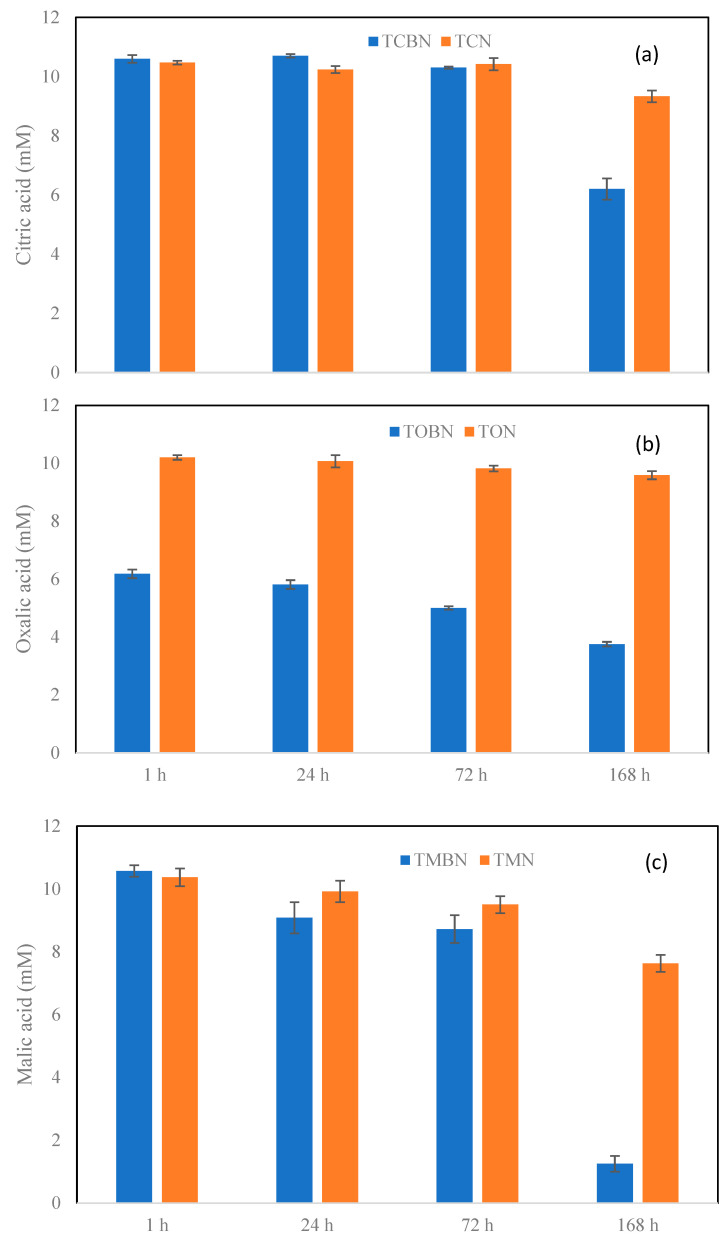
Temporal variation in (**a**) citrate, (**b**) oxalate, and (**c**) malate in the LMWOA only and LMWOA–biochar treatment systems during the period of experiment.

The concentrations of the three types of organic ligands all tended to decrease over time during the period of experiment in the LMWOA only treatments with TMN (malic-acid treatment) showing a clearer decreasing trend for malate. In the LMWOA–biochar systems, a significant decrease in citrate only took place at the 168 h, while a significant drop in oxalate was observed just after the 1 h reaction. Although the concentration of malate decreased slowly prior to the 72 h, it sharply dropped to a very low level (<15% of the theoretical concentration of the added malate) (Figure 1).

### 2.4. Phosphate

In C1 (biochar-only), the concentration of phosphate after the 1 h reaction was 1.39 mg/L and then tended to increase over time. The nitrate-only solution (C2) contained no phosphate. The addition of biochar into the nitrate solution (C3) caused an increase in phosphate in the solution. The combined nitrate and LMWOA solutions contained no phosphate. In all the three biochar–LMWOA–nitrate systems, phosphate was detected with a range of 24.85–53.22 mg/L. However, different temporal variation patterns were observed for different organic-acid treatments with citric- and oxalic-acid treatments showing a trend that phosphate increased over time while the malic-acid treatment showed the opposite (Table 3).

## 3. Materials and Methods

### 3.1. The Biochar Material Used in the Experiment

The biochar was purchased from the Liaoning Golden Future Agriculture Technology Co., Ltd. According to the supplier, this material was produced from rice straw at a pyrolysis temperature of 600 °C with a holding time of 2 h. Prior to the experiment, the biochar material was crushed using a pestle and a mortar and passed through a 2 mm sieve. The biochar had a pH nearly 10 and a BET surface area of 37.85 m^2^/g. Detailed physiochemical characteristics are given in Table 4 and an SEM image of the biochar material is provided in Figure S1 from Supplementary Materias.

### 3.2. Experimental Design

Three controls (C1, C2, and C3) and six LMWOA treatments were set for the experiment. The controls contained no LMWOA with C1 having added biochar only, C2 having added nitrate (using analytical NaNO_3_) only, and C3 having both added biochar and nitrate. There were two sets of LMWOA treatments with the first set containing no added biochar and the second set having the added biochar. Details on the experimental set-up can be seen from Table 5.

The ingredients for each control or treatment were placed in a 150 mL plastic bottle. The batch reactors were shaken in a rotary shaker for 1 h. After shaking, the redox potential (Eh), electrical conductivity (EC), and pH of the solutions were measured, followed by the collection of 5 mL of sample from each bottle. The solution samples were kept at −40 ℃ prior to chemical analysis. After 1 h of shaking, the batch reactors were allowed to stand in dark during the entire period of experiment except for intermittent Eh, EC, and pH measurements and sample collection at the 24, 72, and 168 h of the experiment.

### 3.3. Analytical Methods

The EC, Eh, and pH in the solutions were measured using a calibrated EC meter (DDSJ-308A, Shanghai, China), Eh meter (Thermo Scientific, Waltham, MA, USA), and pH meter (SX-620, Shanghai, China), respectively. The concentrations of NO_2_^−^ and NO_3_^−^ and PO_4_^3−^ in various aqueous samples were determined by ion chromatography (DIONEX ICS-900, Dionex, Waltham, MA, USA). IonPac^®^ AS 20 anion analytical column (4 mm × 250 mm, Dionex, Waltham, MA, USA), IonPac^®^ AG20 guard column (4 mm × 50 mm, Dionex, Waltham, MA, USA), and AERS anion self-regenerating suppressor 500 (4 mm, Dionex, USA) were used. A mixture of 30 mM sodium carbonate (KOH, Guangzhou, China) and ultrapure water (18.2 MΩ /cm, Guangzhou, China) was used as the mobile phase. The flow rate was set at 1.0 mL/min with 20 μL injection volume. Various low-molecular-weight organic ligands in the solutions were determined by ionic chromatography (IC-900, Dionex, Waltham, MA, USA) using an IonPac ICE-AS6 ion exclusion column (9 mm × 250 mm, Dionex, Waltham, MA, USA).

### 3.4. Quality Assurance and Quality Control

The experiments were performed in triplicate. Repeatability analysis shows that the mean RSDs for Eh, EC, pH, NO_3_^−^, PO_4_^3−^, citrate, oxalate, and malate were 0.96%, 2.41%, 1.71, 3.72%, 3.29%, 2.78%, 1.55%, and 5.44%, respectively.

### 3.5. Statistical Analysis

Statistical difference analysis was performed using IBM SPSS software Version 22.0. The experimental data were analyzed by one-way analysis of variance (ANOVA), and the means were compared using the significant difference (Duncan) method at 5% level.

## 4. Discussion

**Figure 2:**
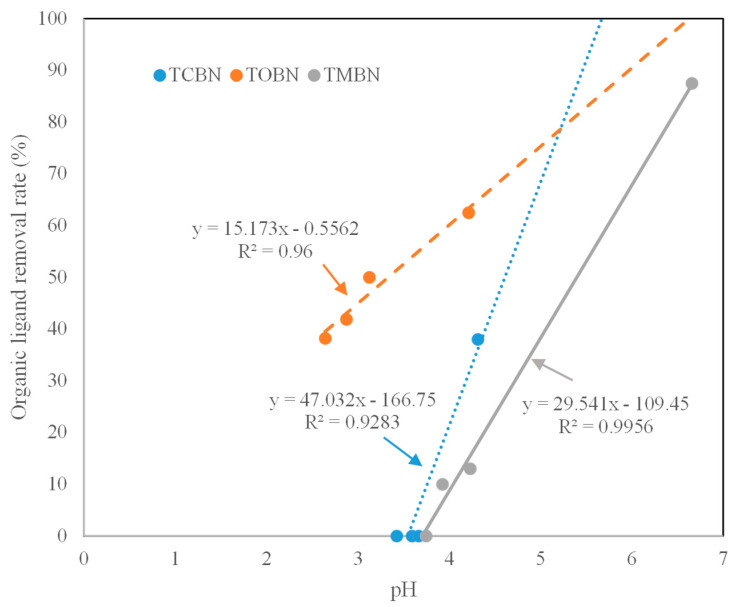
Relationship between pH and organic ligand removal rate in the three biochar−LMWOA−nitrate systems.

The gentle decrease in various solution-borne organic ligands over time in the LMWOA–nitrate systems suggests that decomposition of organic ligands took place during the period of the experiment. The solutions were not sterilized so it was likely that the degradation of these LMWOA ligands was mediated by microbes [34]. The more significant removal of solution-borne LMWOA ligands in the biochar–LOWOA–nitrate systems compared to the LMWOA–nitrate systems suggests adsorption of LMWOA ligands by biochar took place. It is interesting to note that the removal rates of all the three organic ligands increased with increasing pH (Figure 2), suggesting strong control of organic ligand adsorption by solution pH. Citric, oxalic, and malic acids are weak acids, which only partially dissociate in solutions, resulting in the co-presence of different organic ligand species [35]. Prior to the 72 h of the experiment, the pH in the solution was around 3.5 when the citrate was predominantly in the form of dihydrogen citrate ion (H_2_Cit^−^), which might not be sufficiently strong to compete with other anions for the available adsorption sites on the biochar surfaces. The increase in pH to above 4 at the 168 h of the experiment allowed a shift of dominant citrate species from H_2_Cit^−^ to monohydrogen citrate ion (HCit^2−^) that has a stronger affinity to protonated biochar surfaces because the affinity of an anion towards an adsorption site tends to increase with increasing valency of that anion [24]. Similarly, the pH increase from about 2.6 to 4.2 resulted in a shift of dominant oxalate ion species from monohydrogen oxalate ion (HC_2_O_4_^−^) to oxalate ion (C_2_O_4_^2−^). The extremely high removal rate of malate at the 168 h of the experiment was attributable to the nearly circumneutral pH, which made the malate ion species almost completely in the form of malate ion (C_4_H_4_O_5_^2−^) that had the highest affinity to the biochar adsorption sites compared to any other malate ion species.

Biochar and LMWOAs have the potential to donate electrons and cause the reduction of some chemical compounds [7]. However, the absence of nitrite in any controls and treatments in this study appears to suggest that the added biochar or/and LMWOAs were not capable of causing the reduction of the added nitrate despite that the pH and Eh values in the investigated systems were outside the pH-Eh stability field for nitrate [36].This was probably because there was not a sufficient amount of nitrate-reducing microbes present in the systems. For this reason, the removal of nitrate from the solutions can be solely attributed to adsorption by the biochar. The possible adsorption mechanisms may include:

[PB]^+^ + NO_3_^−^ → [PB]^+^−NO_3_^−^ (1)

[PB]^+^ + OL^2−^ + M^2+^ + NO_3_^−^ → [PB-OL]^−^−MNO_3_^+^ (2)

In the above chemical equations, PB denotes protonated biochar; OL stands for an organic ligand; and M represents a metal. In Equation (1), nitrate ion was directly adsorbed to the biochar surface when there was no competition for the adsorption site from an organic ligand. In Equation (2), a divalent anionic ligand was adsorbed to the biochar surface, creating a negatively charged site on the new surface, which allowed binding of a divalent cation nitrate complex (MNO_3_^+^). The biochar used in this study contained soluble Ca at a concentration of 0.31 g/kg (Table 1). It is also possible that LMWOAs could solubilize biochar-borne minerals, such as aluminuim, calcium, and iron compounds [26]. The Ca^2+^ entered into the solutions from the biochar allowed formation of CaNO_3_^+^ that could be adsorbed to the negatively charged site on the new biochar surfaces, as described in Equation (2). Qin et al. [9] showed that soluble Ca^2+^ acted as a bridge linking negatively charged biochar surfaces and anionic nitrate during biochar-driven adsorption of soluble nitrate under the non-acidic condition. Under acidic conditions, such as the current reaction systems, cationic aluminium and iron species, such as Al^3+^, Al(OH)^2+^, Fe^3+^, and Fe(OH)^2+^ could also form ionic complexes with nitrate, which were then adsorbed by the negatively charged biochar surfaces, e.g.,

[PB-OL]^−^ + Al(NO_3_)_2_^+^ → [PB-OL]^−^−Al(NO_3_)_2_^+^ (3)

[PB-OL]^−^ + Fe(NO_3_)_2_^+^ → [PB-OL]^−^−Fe(NO_3_)_2_^+^ (4)

[PB-OL]^−^ + Al(OH)(NO_3_)^+^ → [PB-OL]^−^−Al(OH)(NO_3_)^+^ (5)

[PB-OL]^−^ + Fe(OH)(NO_3_)^+^ → [PB-OL]^−^−Fe(OH)(NO_3_)^+^ (6)

**Figure 3:**
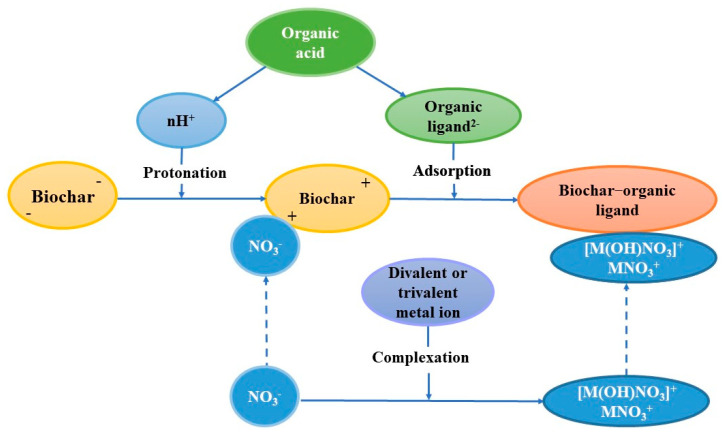
A schematic flow chart illustrating the chemical mechanisms responsible for the nitrate retention by biochar in the presence of LMWOAs.

The overall chemical mechanisms responsible for the observed removal of solution-borne nitrate in this study are illustrated in Figure 3. Although the protonation of biochar surfaces allows direct adsorption of NO_3_^−^ onto the biochar surfaces, the negatively organic ligands can compete with NO_3_^−^ for the available protonated biochar surfaces, which then acts as a bridge to bind cationic metal–nitrate complexes. This creates an additional avenue for the immobilization of solution-borne NO_3_^−^ in the combined biochar–LMWOA systems.

**Figure 4:**
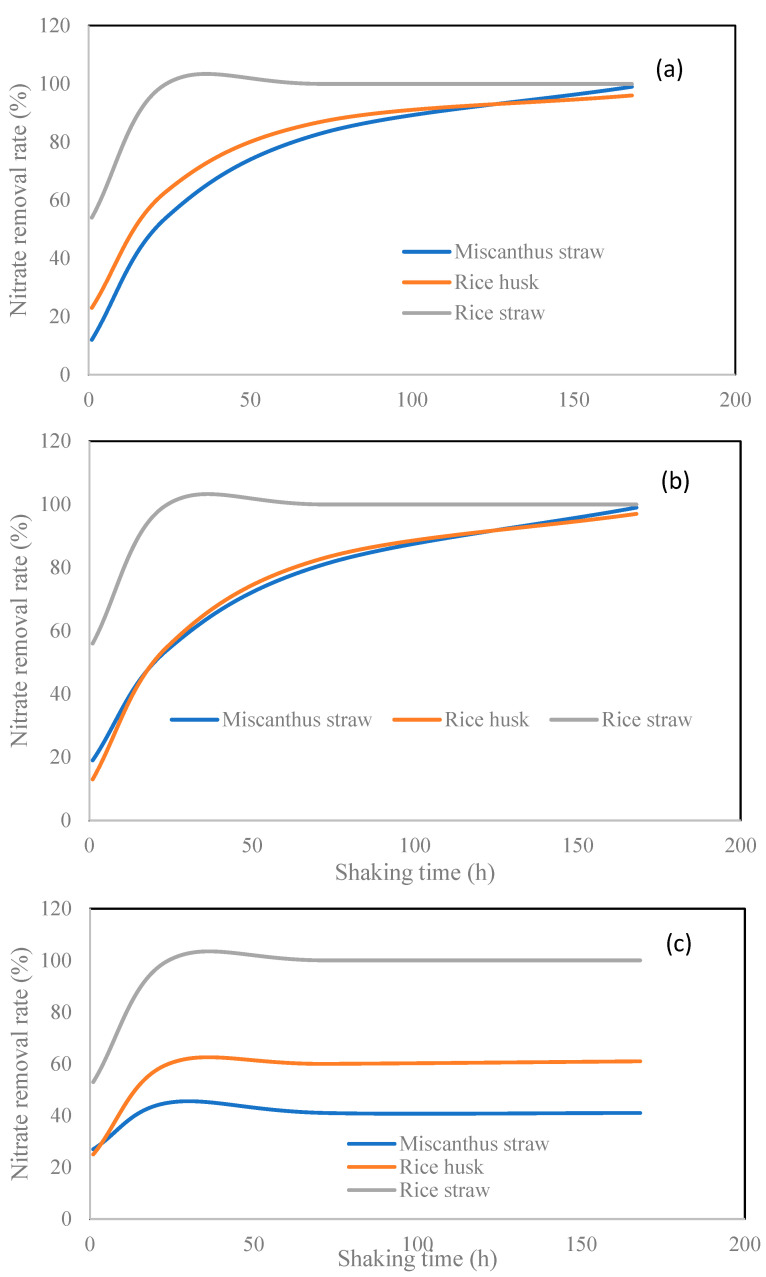
A comparison of nitrate removal by three different biochar materials in the presence of (**a**) citric acid, (**b**) oxalic acid, and (**c**) malic acid (data on the miscanthus and rice husk biochars were from Heaney et al. [11]).

The complete disappearance of solution-borne nitrate at the 24 h of the experiment suggests the adsorption reactions possibly via Equations (1)–(6) were very rapid under the experimental conditions set in this study. The concentration of LMWOAs in this study was lower (0.01 M) compared to that (0.02 M) in Heaney et al. [11]. In theory, the use of more acidic solutions allows stronger protonation of the biochar surfaces. However, it is surprising that the removal efficiency of nitrate in Heaney et al. [11] was much lower than in the current study. In all the three LMWOA treatment systems, the added nitrate in the current study was completely removed from the solution just after the 1 h reaction, while it required 168 hours to achieve a similar outcome for the citric- and oxalic-acid treatments in Heaney et al. [11]. For the malic-acid treatment, the nitrate-removal rates for the biochars used in Heaney et al. [11] were only approximately 40% and 60%, respectively (Figure 4). This suggests that a higher degree of biochar surface protonation does not necessary enhance nitrate adsorption. In addition, other factors may play important roles in the nitrate adsorption in the combined biochar and LMWOA systems. The biochar materials used in Heaney et al. [11] were produced from miscanthus straw and rice husks with pyrolysis temperature of 700 °C. This difference in biochar types may be partially responsible for the observed differential nitrate removal efficiency. However, it appears that the more acidic conditions formed under the higher dose of LMWOA played a more important role in adversely affecting the nitrate-removal efficiency. Under more acidic conditions, the LMWOA ligand was likely to increase in the single valent form of ligands, such as H_2_Cit^−^, HC_2_O_4_^−^, and C_4_H_5_O_5_^−^, which failed to create the negatively charged new biochar surface, as shown below:

[PB]^+^ + OL^−^ → [PB-OL]^°^ (7)

This means that the adsorption mechanism via Equation (2) was not well operative under more acidic conditions with the increased LMWOA dose in Heaney et al. [11].

The detection of small amounts of phosphate in C1 suggests that some biochar-borne phosphate was in water soluble forms, which released gradually from biochar upon contact with water. The addition of nitrate into the solution (C2) enhanced the release of biochar-borne phosphate, suggesting the possible replacement of adsorbed phosphate by the added nitrate. The addition of LMWOAs caused a significant release of biochar-borne phosphate because LMWOAs are capable of solubilizing phosphate minerals, such as aluminium phosphate, calcium phosphate, and iron phosphate [24,25]. It is interesting to note that phosphate in the malic-acid treatment behaved differently from that in the citric- and oxalic-acid treatments; the former tended to decrease over time while the opposite was observed for the latter. It is worthwhile to note that the solution pH increased over time for these systems (Table 3), which might cause re-precipitation of aluminium and iron phosphates [37]. For the citric- and oxalic-acid treatments, the pH was probably not sufficiently low to cause re-immobilization of phosphate. In comparison, the pH in the malic-acid treatment was significantly higher compared to the other two organic-acid treatments with a pH as high as 6.6 being recorded at the 168 h of the experiment. This explains the re-immobilization of phosphate in the malic acid treatment.

The release of phosphate from the biochar in the presence of LMWOAs suggests that the adsorption of phosphate ions by the protonated biochar surfaces was insignificant. Unlike NO_3_^−^ that can form cationic metal complexes (e.g., Ca NO_3_^+^), PO_4_^3−^ is not capable of forming any cationic phosphate complexes with divalent or trivalent cations. Consequently, the binding of phosphate via an organic ligand bridge, as what took place for nitrate in Equations (2)–(6), was not possible. This means that the dissolved phosphate from the dissolution of biochar-borne metal phosphates did not compete with nitrate for the available binding sites resulting from the adsorption of organic ligands onto biochar surfaces. The phosphate ions in the current reaction systems did not affect nitrate retention during the experiment, as confirmed by the observation that the desorption of nitrate did not occur despite the presence of soluble phosphate in the solutions.

## 5. Conclusions

Under the set experimental conditions in this study, the adsorption of LMWOA ligands by the biochar was enhanced with increasing pH. Nitrate ion could be directly adsorbed to the protonated biochar surface when there was no competition from organic ligands for the available adsorption sites. Divalent organic ligands could be adsorbed to the biochar surface and thus create negatively charged sites to allow binding of cationic metal nitrate complexes. A higher degree of biochar surface protonation does not necessarily enhance nitrate adsorption, and other factors may play important roles in the nitrate adsorption in combined biochar and LMWOA systems. More acidic conditions formed under the higher dose of LMWOA played a more important role in adversely affecting the nitrate-removal efficiency. LMWOAs caused significant release of biochar-borne phosphate. The phosphate in the malic-acid treatment tended to decrease over time, while the opposite was observed for that in the citric- and oxalic-acid treatments. This was caused by re-immobilization of phosphate in the former due to the marked increase in solution pH.

## Figures and Tables

**Table 1:** Eh, EC, and pH measured at different times during the experiment for the controls and treatments.

Parameter	Treatment	1 h	24 h	72 h	168 h
Eh	C1	481.00 ± 30.36 (a) [A]	525.00 ± 6.30 (a) [A]	492.03 ± 0.93 (d) [A]	346.80 ± 0.75 (f) [B]
	C2	428.57 ± 6.72 (c) [B]	468.77 ± 3.46 (d) [A]	414.13 ± 4.39 (f) [B]	428.63 ± 1.60 (d) [B]
	C3	437.43 ± 2.45 (bc) [B]	500.00 ± 1.55 (c) [A]	445.47 ± 4.54 (e) [B]	372.97 ± 3.22 (e) [C]
	TCN	576.57 ± 0.72 (a) [A]	513.57 ± 2.17 (b) [C]	521.67 ± 1.78 (a) [B]	470.17 ± 0.78 (ab) [D]
	TON	578.70 ± 13.73 (a) [A]	529.37 ± 1.65 (a) [B]	528.70 ± 0.44 (a) [B]	472.87 ± 1.63 (ab) [C]
	TMN	539.87 ± 2.20 (bc) [A]	529.50 ± 4.24 (a) [AB]	523.40 ± 3.98 (a) [B]	463.13 ± 2.24 (abc) [C]
	TCBN	527.73 ± 0.81 (c) [A]	507.03 ± 1.21 (bc) [C]	511.30 ± 1.27 (b) [B]	462.20 ± 0.17 (abc) [D]
	TOBN	551.47 ± 1.23 (b) [A]	503.50 ± 1.00 (c) [C]	509.77 ± 0.07 (b) [B]	463.50 ± 0.45 (abc) [D]
	TMBN	527.10 ± 1.28 (c) [A]	501.23 ± 0.28 (c) [B]	508.97 ± 0.38 (b) [B]	452.30 ± 6.43 (abc) [C]
EC	C1	0.33 ± 0.00 (f) [B]	0.30 ± 0.00 (cd) [C]	0.33 ± 0.00 (cd) [B]	0.35 ± 0.00 (c) [A]
	C2	0.09 ± 0.00 (g) [A]	0.07 ± 0.01 (d) [AB]	0.08 ± 0.00 (d) [AB]	0.08 ± 0.00 (c) [AB]
	C3	0.39 ± 0.00 (e) [B]	0.36 ± 0.00 (cd) [C]	0.39 ± 0.00 (cd) [B]	0.41 ± 0.00 (c) [A]
	TCN	0.75 ± 0.00 (c) [A]	0.64 ± 0.00 (bc) [B]	0.63 ± 0.01 (bc) [B]	0.57 ± 0.01 (bc) [C]
	TON	2.49 ± 0.01 (a) [A]	2.14 ± 0.01 (a) [B]	2.13 ± 0.00 (a) [B]	2.14 ± 0.01 (a) [B]
	TMN	0.61 ± 0.05 (d) [A]	1.08 ± 0.50 (b) [A]	1.07 ± 0.51 (b) [A]	1.05 ± 0.52 (b) [A]
	TCBN	0.76 ± 0.00 (c) [A]	0.68 ± 0.00 (bc) [B]	0.68 ± 0.00 (bc) [B]	0.60 ± 0.00 (bc) [C]
	TOBN	1.38 ± 0.01 (b) [A]	0.93 ± 0.01 (b) [B]	0.73 ± 0.01 (bc) [C]	0.56 ± 0.01 (bc) [D]
	TMBN	0.72 ± 0.01 (c) [A]	0.64 ± 0.00 (bc) [B]	0.64 ± 0.00 (bc) [B]	0.61 ± 0.00 (bc) [C]
pH	C1	8.55 ± 0.01 (a) [D]	8.92 ± 0.05 (a) [C]	9.40 ± 0.01 (a) [B]	9.82 ± 0.00 (a) [A]
	C2	7.03 ± 0.04 (b) [B]	7.23 ± 0.04 (c) [A]	6.50 ± 0.05 (c) [C]	6.91 ± 0.01 (b) [B]
	C3	8.54 ± 0.01 (a) [D]	8.70 ± 0.05 (b) [C]	9.12 ± 0.08 (b) [B]	9.77 ± 0.04 (a) [A]
	TCN	2.67 ± 0.01 (e) [B]	2.66 ± 0.00 (g) [B]	2.62 ± 0.00 (g) [C]	2.74 ± 0.01 (e) [A]
	TON	2.19 ± 0.01 (f) [A]	2.18 ± 0.01 (h) [A]	2.13 ± 0.01 (h) [B]	2.19 ± 0.01 (f) [A]
	TMN	2.54 ± 0.17 (e) [A]	2.56 ± 0.16 (g) [A]	2.48 ± 0.17 (g) [A]	2.54 ± 0.17 (e) [A]
	TCBN	3.42 ± 0.01 (d) [D]	3.59 ± 0.01 (e) [C]	3.67 ± 0.01 (e) [B]	4.31 ± 0.05 (d) [A]
	TOBN	2.64 ± 0.01 (e) [D]	2.87 ± 0.01 (f) [C]	3.12 ± 0.03 (f) [B]	4.21 ± 0.10 (d) [A]
	TMBN	3.75 ± 0.01 (c) [C]	3.92 ± 0.01 (d) [BC]	4.23 ± 0.07 (d) [B]	6.66 ± 0.26 (c) [A]

All values are presented as mean ± standard error (*n* = 3). Means with different small letters (in round brackets) in the same column for each parameter (i.e., Eh, EC, or pH) are significantly different at *p* < 0.05. Means with different capital letters (in square brackets) in the same row are significantly different at *p* < 0.05.

**Table 2:** Nitrite and nitrate in the solutions collected at different times during the experiment for the controls and treatments.

Parameter	Organic Acid	1 h	24 h	72 h	168 h
NO_2−_	C1	n.d	n.d	n.d	n.d
(mg/L)	C2	n.d	n.d	n.d	n.d
	C3	n.d	n.d	n.d	n.d
	TCN	n.d	n.d	n.d	n.d
	TON	n.d	n.d	n.d	n.d
	TMN	n.d	n.d	n.d	n.d
	TCBN	n.d	n.d	n.d	n.d
	TOBN	n.d	n.d	n.d	n.d
	TMBN	n.d	n.d	n.d	n.d
NO_3__−_	C1	0.52 ± 0.09 (d) [B]	1.76 ± 0.07 (c) [A]	1.83 ± 0.15 (e) [A]	1.83 ± 0.04 (f) [A]
(mg/L)	C2	61.92 ± 0.49 (b) [C]	63.70 ± 0.68 (ab) [B]	65.36 ± 0.34 (b) [A]	65.61 ± 0.26 (a) [A]
	C3	65.96 ± 0.65 (a) [A]	64.55 ± 0.80 (a) [A]	61.53 ± 0.31 (c) [B]	60.92 ± 0.18 (c) [B]
	TCN	60.58 ± 1.16 (b) [A]	60.69 ± 2.18 (ab) [A]	55.49 ± 0.30 (d) [B]	53.26 ± 0.37 (e) [B]
	TON	60.82 ± 2.67 (b) [A]	63.17 ± 0.24 (ab) [A]	61.00 ± 0.39 (c) [A]	62.93 ± 0.10 (b) [A]
	TMN	59.22 ± 0.27 (b) [B]	63.01 ± 1.65 (ab) [A]	66.42 ± 0.80 (a) [A]	59.35 ± 1.16 (d) [B]
	TCBN	28.57 ± 0.85 (c) [A]	0.00 ± 0.00 (c) [B]	0.00 ± 0.00 (f) [B]	0.00 ± 0.00 (g) [B]
	TOBN	29.21 ± 0.98 (c) [A]	0.00 ± 0.00 (c) [B]	0.00 ± 0.00 (f) [B]	0.00 ± 0.00 (g) [B]
	TMBN	27.09 ± 2.24 (c) [A]	0.00 ± 0.00 (b) [B]	0.00 ± 0.00 (f) [B]	0.00 ± 0.00 (g) [B]

n.d: no detectable. All values are presented as mean ± standard error (*n* = 3). Means with different small letters (in round brackets) in the same column for each parameter (i.e., NO_2−_ or NO_3−_) are significantly different at *p* < 0.05. Means with different capital letters (in square brackets) in the same row are significantly different at *p* < 0.05.

**Table 3:** Phosphate in the solutions collected at different times during the experiment for the controls and treatments.

Parameter	Organic Acid	1 h	24 h	72 h	168 h
PO_4_^3−^	C1	1.39 ± 0.15 (e) [B]	1.66 ± 0.15 (c) [B]	2.39 ± 0.12 (e) [A]	2.40 ± 0.08 (e) [A]
(mg/L)	C2	0.00 ± 0.00 (e)	0.00 ± 0.00 (b)	0.00 ± 0.00 (f)	0.00 ± 0.00 (f)
	C3	6.45 ± 0.09 (d) [B]	6.42 ± 0.18 (a) [B]	7.34 ± 0.21 (d) [A]	6.95 ± 0.32 (d) [AB]
	TCN	0.00 ± 0.00 (e)	0.00 ± 0.00 (c)	0.00 ± 0.00 (f)	0.00 ± 0.00 (f)
	TON	0.00 ± 0.00 (e)	0.00 ± 0.00 (c)	0.00 ± 0.00 (f)	0.00 ± 0.00 (f)
	TMN	0.00 ± 0.00 (e)	0.00 ± 0.00 (c)	0.00 ± 0.00 (f)	0.00 ± 0.00 (f)
	TCBN	40.71 ± 0.70 (b) [C]	41.97 ± 1.49 (a) [C]	46.35 ± 0.56 (b) [B]	51.00 ± 0.16 (b) [A]
	TOBN	37.96 ± 0.94 (c) [C]	41.84 ± 2.22 (a) [C]	48.70 ± 0.35 (a) [B]	53.22 ± 0.41 (a) [A]
	TMBN	44.27 ± 0.89 (a) [A]	41.17 ± 0.25 (c) [A]	35.73 ± 2.04 (c) [B]	24.85 ± 1.10 (c) [C]

All values are presented as mean ± standard error (*n* = 3). Means with different small letters (in round brackets) in the same column are significantly different at *p* < 0.05. Means with different capital letters (in square brackets) in the same row are significantly different at *p* < 0.05.

**Table 4:** Major physiochemical characteristics of the biochar in the experiment.

Parameter	Biochar
pH	9.94
EC (dS/m)	8.05
BET surface area (m^2^/g)	37.85
TOC (g/kg)	420
Soluble Ca (g/kg)	0.31
Soluble Mg (g/kg)	0.06
Soluble Na (g/kg)	0.69
Total N (g/kg)	7.9
Total P (mg/kg)	2.35
Available P (mg/kg)	59.23
Available K (g/kg)	9.65

**Table 5:** Details on water solution experimental set-up.

Treatment	Organic Acid	Biochar (g)	NO_3_^−^ (mM)	Solution (mL)
C1	No	1.0	0.0	100 mL
C2	No	0.0	1.0	100 mL
C3	No	1.0	1.0	100 mL
TCN	Citric	0	1.0	100 mL
TON	Oxalic	0	1.0	100 mL
TMN	Malic	0	1.0	100 mL
TCBN	Citric	1.0	1.0	100 mL
TOBN	Oxalic	1.0	1.0	100 mL
TMBN	Malic	1.0	1.0	100 mL

## Data Availability

Not applicable.

## Supplementary Materials

The following supporting information can be downloaded at: https://www.mdpi.com/article/10.3390/molecules27185811/s1, Figure S1: SEM image of the biochar used in the experiment.
