# Supplementary material for: Behaviors of Organic Ligands and Phosphate during Biochar-Driven Nitrate Adsorption in the Presence of Low-Molecular-Weight Organic Acids
Source: Molecules. 2022 Sep 8;27(18):5811. doi: 10.3390/molecules27185811 (PMC9505611; doi:10.3390/molecules27185811)
Supplement: Supplementary file 1 [file molecules-27-05811-s001.zip › molecules-1843787-supplementary.pdf]

Supplementary Materials

Behaviors of Organic Ligands and Phosphate during Biochar-Driven Nitrate Adsorption in the Presence of Low-Molecular-Weight Organic Acids

Wenming Xiong [1], **Yongjun Li** [1], **Jidong Ying** [2], **Chuxia Lin** [3] and **Junhao Qin** [2,*]

[1] Department, Guangdong Jiangmen Chinese Medicine College, Jiangmen 529000, China
[2] College of Natural Resources and Environment, South China Agricultural University/Key Laboratory of Agro-Environment in the Tropics, Ministry of Agriculture of China, Guangzhou 510642, China
[3] Centre for Regional and Rural Futures, Faculty of Science, Engineering and Built Environment, Deakin University, Burwood, VIC 3125, Australia
* Correspondence: j_qin@scau.edu.cn; Tel.: +86-020-8528078

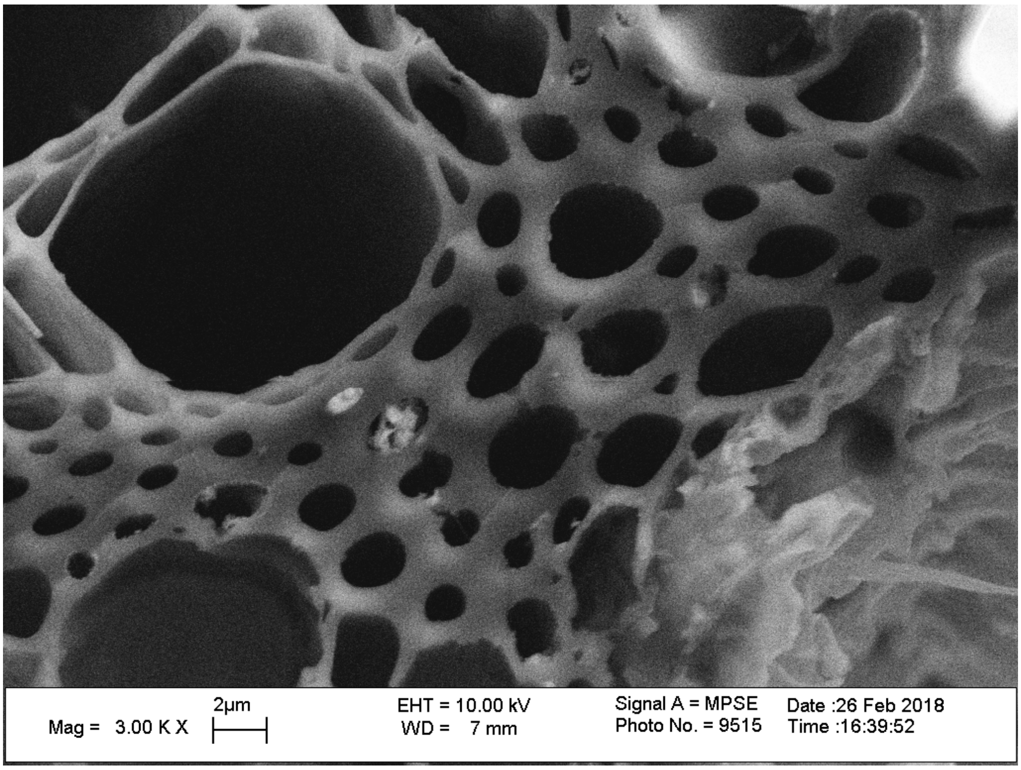

Figure S1. SEM image of the biochar used in the experiment.
